# Hyalinizing clear cell carcinoma in the nasopharynx: A case report and literature review

**DOI:** 10.1097/MD.0000000000039163

**Published:** 2024-08-09

**Authors:** Yu-Wei Chang, Hsing-Mei Wu

**Affiliations:** a Department of Otolaryngology, Shin-Kong Wu-Ho-Su Memorial Hospital, Taipei, Taiwan; b Medical School, Fu-Jen Catholic University, Taipei, Taiwan.

**Keywords:** hyalinizing clear cell carcinoma, metastasis, nasopharynx, salivary gland, surgical resection

## Abstract

**Rationale:**

Hyalinizing clear cell carcinoma (HCCC) arising from a minor salivary gland is a rare malignant neoplasm. Most HCCC has been reported in the palate and tongue base, and only rarely in the nasopharynx. Here, we report a rare case of nasopharyngeal HCCC.

**Patient concerns:**

A 44-year-old male who complained of otorrhea and aural fullness for 5 years was found to have a nasopharyngeal mass.

**Diagnoses:**

HCCC by fluorescence in situ hybridization analysis.

**Interventions:**

Surgical resection plus concurrent chemotherapy and radiation therapy were administered.

**Outcomes:**

The patient recovered well with symptoms improved at postoperative follow-up.

**Lessons:**

HCCC should be included in the differential diagnosis of nasopharyngeal mass. Overall, the prognosis of HCCC is positive after tumor resection and adequate management.

## 1. Introduction

Hyalinizing clear cell carcinoma (HCCC) is a rare, low-grade malignant tumor of the salivary gland, representing <1% of all salivary gland tumors.^[1,2]^ Most HCCC arises from the minor salivary gland in the palate, tongue base, or mouth floor. It has also been reported to arise in the sinonasal cavity, submandibular gland, parotid gland, larynx, and rarely, the nasopharynx.^[2]^ The neck or distant metastasis rate of HCCC is reported to be 15%–25% when the disease is diagnosed, and neck mass lesions are not frequently seen as the initial presentation.^[3]^

The initial presentation of symptoms depends on the tumor location. For example, patients often complain of otorrhea, repeated epistaxis, tinnitus, or nasal congestion when HCCC arises from the nasopharynx.^[2]^

Management of HCCC in the nasopharynx classically includes surgical resection. Although no guidelines are available for indications for radiation therapy, many cases are reported to have undergone radiation therapy to prevent recurrence, especially when negative margins of surgical resection were not achieved.^[4]^ Overall, the prognosis of HCCC is positive after tumor resection and adequate management.

Herein, we report the case of a 44-year-old male who complained of tinnitus and aural fullness for 5 years and was eventually diagnosed with nasopharyngeal HCCC by fluorescence in situ hybridization (FISH) analysis for Ewing sarcoma breakpoint region 1 (ESWR1) fusion genes.

## 2. Case report

**Figure 1.**
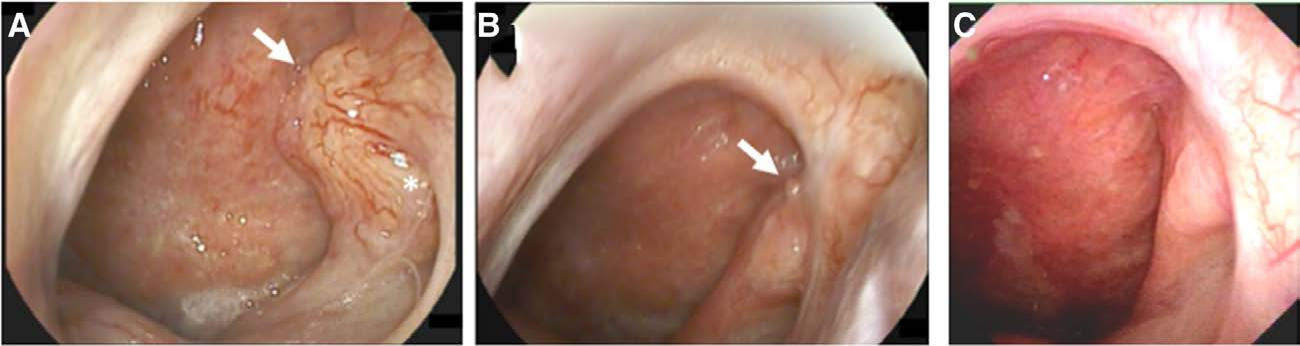
Clinical endoscopic pictures of the hyalinizing clear cell carcinoma in the nasopharynx at preoperation (A), postoperation (B), and under concurrent chemotherapy and radiation therapy (C). The arrowhead indicates the tumor. At preoperation (A), the tumor with a smooth surface located in the left Rosenmüller fossa with extension to the roof of the left Eustachian tube opening (asterisk). The tumor size decreases significantly postoperatively and after concurrent chemotherapy and radiation therapy.

A 44-year-old male who was a hepatitis B virus carrier with a smoking habit and betel nut chewing for >20 years complained of left aural fullness and left tinnitus for 5 years. Medication was administered but the symptoms persisted. Local findings revealed left middle ear effusion with an air bubble. Nasopharyngeal scope examination demonstrated a mass lesion in the left Rosenmüller fossa with extension to the roof of the left Eustachian tube (E-tube) opening (Fig. 1). Biopsy of the lesion was taken. Microscopically, the frozen sections and permanent section showed small fragments of nasopharyngeal tissue with infiltrating carcinoma, comprising monotonous epithelioid cells with eosinophilic to clear cytoplasm, round nuclei, and distinct nucleoli, arranged in a small nesting growth pattern. Pathological examination of the stroma showed focal hyalinization and fibromyxoid changes.

Further immunohistochemical staining was performed for differential diagnosis. The tumor cells were diffusely positive for p40 and CK5/6, focally positive for CK (AE1/AE3) and CK7, while nonreactive for GATA3, SOX10, S100, and neuroendocrine markers. Staining for INI-1 showed intact nuclear expression. Further FISH analysis for ESWR1 fusion genes was positive for break-apart signals, which has been described as a useful tool for HCCC diagnosis.^[4–7]^ Based on these pathologic features and molecular findings, HCCC of salivary gland origin was the favored diagnosis.

**Figure 2.**
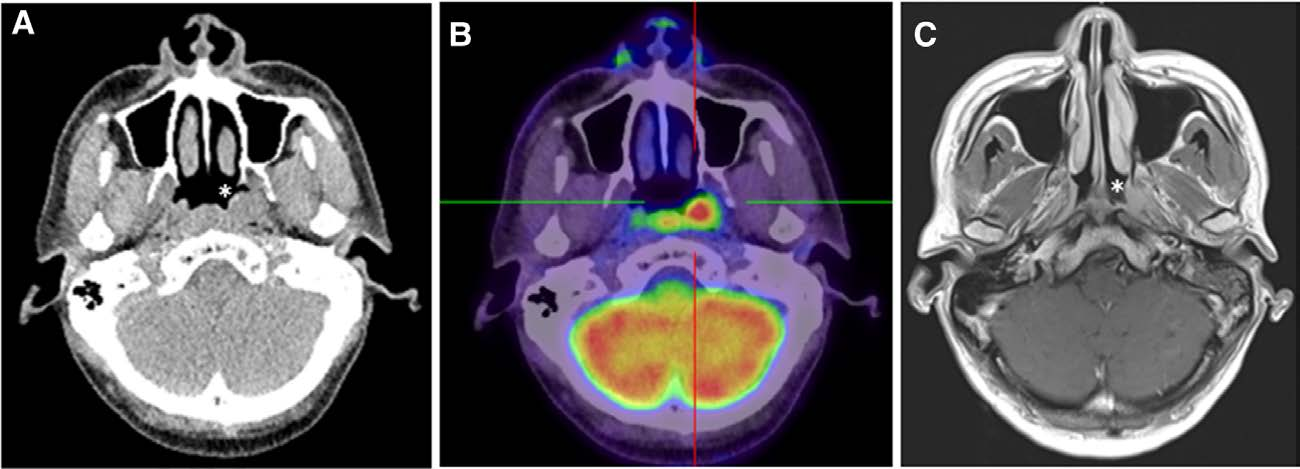
Radiological pictures of the hyalinizing clear cell carcinoma in the nasopharynx. Axial cuts of computed tomography (A), positron emission tomography-computed tomography (B), and magnetic resonance imaging (C) of T1-weighted image with contrast. The asterisk indicates the tumor. In (B), a bulge tumor with intense fluorodeoxyglucose uptake in the left nasopharynx with (1.2 cm, SUV_max_: 14.5) was noted. In (C), the axial cut demonstrates the lateral extension of the tumor to the Eustachian tube.

In addition, magnetic resonance imaging (MRI) demonstrated that the tumor with the largest diameter of 1.3 cm in the nasopharynx, extended laterally to the E-tube (Fig. 2). No obvious nodular lesion was found in the bilateral cervical region. Positron emission tomography-computed tomography scan revealed no distant metastasis. The tentative stage was cT1N0M0, stage I.

**Figure 3.**
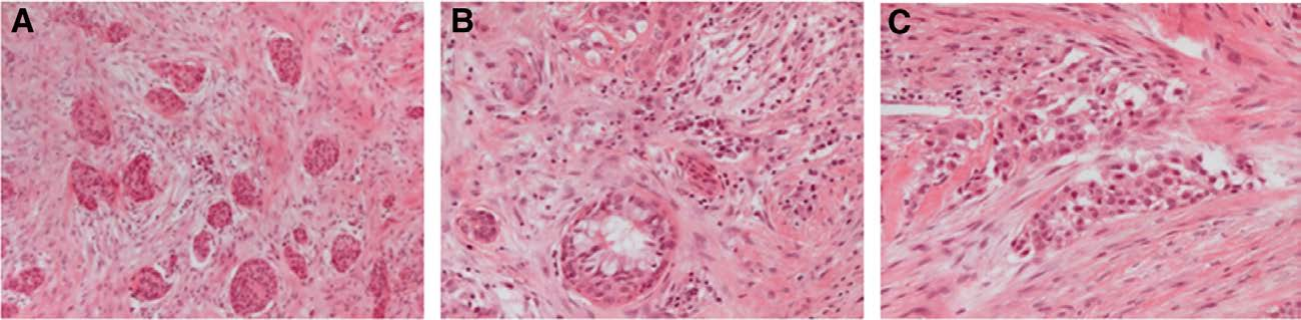
Pathologic specimen photograph. Hematoxylin and eosin stain (×40) (A), hematoxylin and eosin stain (×100) (B), and hematoxylin and eosin stain (×200) (C) show islands of polygonal neoplastic cells displaying round hyperchromatic nuclei, small nucleoli, and acidophilic, or clear cytoplasm. Cells are arranged in irregularly shaped solid islands, infiltrating in sclerotic or hyalinized stroma. Occasional abnormal mitotic figures are seen.

Endoscopic wide excision of the tumor was performed and surgical specimens were sent for pathological examination. The frozen sections of the superior margin and inferior margin showed no evidence of direct tumor invasion. The permanent section pathology, which is more detailed, showed islands of neoplastic cells displaying round hyperchromatic nuclei, small nucleoli, and acidophilic or clear cytoplasm (Fig. 3), which was consistent with findings in the biopsy specimen. HCCC was diagnosed and the resection margins were free after endoscopic resection. Given the diagnosis and following optimal management reported recently, concurrent chemotherapy and radiation therapy with a total radiation dose of 70 Gy were given. The symptoms of aural fullness and tinnitus improved postoperatively, and there was no sign of recurrence during postoperative follow-up.

## 3. Discussion

This article reports a rare case of HCCC in the nasopharynx, which was confirmed by genetic testing of ESWR1 using FISH analysis. Subsequent endoscopic resection was done with a resection margin free of tumor invasion, and further CCRT was performed. No evidence of recurrence was noted during the postoperative follow-up for 1 year.

The most common symptom of nasopharyngeal carcinoma is a cervical mass,^[8]^ whereas neck metastasis is seldom the initial presentation in HCCC.^[3]^ Other common symptoms of nasopharyngeal HCCC are reported to be otorrhea, nasal congestion, and tinnitus.^[2]^ In the present case, the patient complained of left aural fullness and left tinnitus for 5 years. Local findings revealed left middle ear effusion and no palpable mass in the neck, which was compatible with findings in the studies above. MRI revealed no obvious nodular lesion in the bilateral cervical region and positron emission tomography-computed tomography revealed no distant metastasis, which defined the TNM staging as pT1N0M0. This is consistent with previous studies in which distant metastasis was not common in HCCC.^[1]^ Hence, when a patient presents with middle ear effusion and complains of otologic symptoms such as aural fullness or tinnitus, it is necessary to examine the nasopharynx.

Although surgical resection has been considered as first-line treatment for HCCC, no management guidelines are yet available for nasopharyngeal HCCC, and the possible roles of adjuvant radiation therapy and chemotherapy remain unclear. In the absence of actual clinical guidelines, previous case reports show the management trends for HCCC. Fukuda et al^[6]^ reported a 63-year-old female with HCCC in the nasopharyngeal roof with the staging of pT1N0M0. Surgical resection was done and no evidence of recurrence was found after 1 year. Nakashima et al^[9]^ also reported a 27-year-old female with HCCC in the nasopharynx without cervical or distant metastasis. Transoral and transpalatal wide resections were done and no evidence of recurrence was found after 1 year. Cheng et al^[10]^ reported a 63-year-old female with nasopharyngeal HCCC extended to the oropharynx and choana, without neck or distant metastasis. Endoscopic resection was done and radiation therapy was also performed. No evidence of recurrence was found after 1 year. Ceballos Saenz et al^[4]^ reported a 38-year-old female with HCCC in the nasopharynx invading into the skull base and the right foramen ovale. Surgical resection plus adjuvant radiotherapy and chemotherapy were done, and no evidence of recurrence was documented. In the present case, the resection margin was free of tumor invasion in both the frozen section and permanent section pathology examination. However, the skull base and E-tube were left intact during the endoscopic resection. Therefore, possible invasion of the tumor at the skull base or E-tube could not be ruled out, particularly since the MRI revealed the lateral extension of the tumor to the E-tube, suggesting concurrent chemotherapy and radiation therapy. Radiation therapy with a total dose of 70 Gy targeting the nasopharynx, E-tube, and bilateral neck was performed to prevent tumor invasion.

To the best of our knowledge, this is the first case of HCCC in the nasopharynx extended to the E-tube diagnosed by FISH analysis for ESWR1 fusion genes in Taiwan. However, it is limited by the rarity of HCCC cases, especially HCCC arising from the nasopharynx, as well as the lack of clinical guidelines for the management of HCCC. Although the prognosis of HCCC is favorable after surgical resection and mortality has been reported in only one previous case,^[11]^ follow-up of the present case was only 1 year. Long-term prognosis after tumor resection with/without radiotherapy or chemotherapy should be determined through regularly scheduled follow-up, including symptoms, endoscope examination, and radiological studies. Last, although surgical resection plus radiation therapy for HCCC has been documented in previous studies,^[2,3,12]^ few studies have reported administering chemotherapy.^[4]^ In the present case, concurrent chemotherapy was administered to help prevent possible neck metastasis and tumor invasion in the skull base and E-tube.

## 4. Conclusion

HCCC arising from a minor salivary gland in the nasopharynx is uncommon. Surgical resection with adequate margin seems the main treatment for minor salivary gland tumor, different from nasopharyngeal carcinoma. Further studies with more patients are needed to establish the roles of adjuvant radiotherapy or chemotherapy for HCCC.

A better understanding of the biology of the tumor is also warranted for standard treatment. Clinical guidelines are greatly needed to help physicians diagnose, treat, and manage HCCC.

## Author contributions

**Conceptualization:** Hsing-Mei Wu.

**Data curation:** Yu-Wei Chang, Hsing-Mei Wu.

**Project administration:** Yu-Wei Chang, Hsing-Mei Wu.

**Supervision:** Hsing-Mei Wu.

**Validation:** Hsing-Mei Wu.

**Writing—original draft:** Yu-Wei Chang, Hsing-Mei Wu.

**Formal analysis:** Hsing-Mei Wu.

**Investigation:** Hsing-mei Wu.

**Writing—review and editing:** Hsing-mei Wu.
